# Interaction between physical activity, *PITX1* rs647161 genetic polymorphism and colorectal cancer risk in a Korean population: a case-control study

**DOI:** 10.18632/oncotarget.24136

**Published:** 2018-01-10

**Authors:** Madhawa Neranjan Gunathilake, Jeonghee Lee, Young Ae Cho, Jae Hwan Oh, Hee Jin Chang, Dae Kyung Sohn, Aesun Shin, Jeongseon Kim

**Affiliations:** ^1^ Department of Cancer Control and Population Health, Graduate School of Cancer Science and Policy, Goyang-si, Gyeonggi-do 10408, South Korea; ^2^ Department of Biomedical Science, Graduate School of Cancer Science and Policy, Goyang-si, Gyeonggi-do 10408, South Korea; ^3^ Center for Colorectal Cancer, National Cancer Center Hospital, National Cancer Center, Goyang-si, Gyeonggi-do 10408, South Korea; ^4^ Department of Preventive Medicine, Seoul National University College of Medicine, Jongno-gu, Seoul 03080, South Korea

**Keywords:** physical activity, PITX1, genetic polymorphism, colorectal cancer, interaction

## Abstract

This study assessed the interaction between physical activity and colorectal cancer (CRC) risk based on a polymorphism in the paired-like homeodomain 1 (*PITX1*) gene in Koreans. In total, 923 cases and 1,846 controls were enrolled at the National Cancer Center, Korea. Subjects who did regular exercise showed a significantly reduced risk of CRC than those did not exercise regularly (OR = 0.37, 95% CI = 0.30–0.45). Subjects in the highest tertile of metabolic equivalents of task (MET)-minutes per week showed a significantly lower risk of CRC (OR = 0.62, 95% CI = 0.48–0.79, p-trend < 0.001). In the dominant model, minor allele carriers showed a significantly higher risk of CRC than subjects homozygous for the major allele (OR = 1.46, 95% CI = 1.18–1.80). The PITX1 genetic variant showed significant interactions with regular exercise and CRC risk (p-interaction = 0.018) and colon cancer risk (p-interaction = 0.029) among all subjects. Subjects who carried at least one minor allele and did not regularly exercise showed a greater risk of CRC (OR = 1.81, 95% CI = 1.37–2.41). Subjects who were homozygous for the major allele with high physical activity showed a significantly reduced risk of CRC (OR = 0.56, 95% CI = 0.38–0.82). Thus, individuals with *PITX1* genetic variants can have benefit from physical activity regarding prevention of CRC risk in a Korean population.

## INTRODUCTION

Colorectal cancer (CRC) has emerged as a complex multi-factorial disease. The incidence of CRC continues to increase with the development of technology and the adoption of a more Western lifestyle. Moreover, although CRC is more common in high-income countries, its incidence is currently increasing in middle- and low-income countries [1]. In 2012, GLOBOCAN estimated that CRC is the third most common cancer worldwide, with approximately 1.4 million newly diagnosed cases, and noted that the age standardized rates of CRC in Korea were 45.0 per 100,000 for both sexes and 58.7 and 33.3 per 100,000 for males and females, respectively [2]. However, the Korean Central Cancer Registry data indicated that the CRC incidence decreased in men and stabilized in women in 2013 [3]. The age standardized incidence rate of CRC was 31.9 per 100,000 for both sexes and 42.6 and 23.0 per 100,000 for males and females respectively [4].

CRC is a multifactorial disease with a set of genetic and environmental factors [5]. Several factors, such as lifestyle, nutrition, physical activity, and genetics, play a major role in CRC [6]. Specifically, the role of physical activity in CRC susceptibility has been of interest [6]. The World Cancer Research Fund report, *Food, Nutrition, Physical activity, and the Prevention of Cancer: A Global Perspective*, - identified physical activity as an important factor for decreasing CRC risk [7]. Approximately 13%–14% of CRC cases may be attributed to physical inactivity [7]. Regarding non-modifiable factors, genetics have been considered the most prioritized antecedent for CRC carcinogenesis. According to the genome-wide association studies (GWAS), paired-like homeodomain 1 (*PITX1*) genetic polymorphism has been newly identified as a CRC susceptible genetic factor specifically for the East Asians [8]. Thus, the role of the paired-like homeodomain 1 (*PITX1*) gene should be addressed in CRC.

*PITX1*, which is a bicoid-related homeodomain factor, is a known transcription factor that is expressed in the developing anterior pituitary gland [9, 10]. *PITX1* rs647161 is located on chromosome 5 in region 5q31.1, where a cluster of single nucleotide polymorphisms (SNPs) is associated with CRC carcinogenesis [11]. Of the genes in this region, *PITX1* is the closest to rs647161 (approximately 129 kb upstream) [12]. *PITX1*, which is most likely involved in CRC carcinogenesis, has been considered a tumor suppressor gene [13]. However, the polymorphism reported to have the greatest risk, i.e., rs647161, exhibits an unclear function and is not present in any known transcribed or regulatory sequences [13]. Although rs647161 has an unclear function, genome-wide association studies (GWAS) have recently associated the rs647161 (5q31.1) SNP with the risk of CRC [14, 15]. *PITX1* and other members of the family are involved in embryonic development [16]. Furthermore, these genes control the differentiation and proliferation of mature tissues and can play a causal role in carcinogenesis as tumor suppressor genes [17].

*PITX1* expression influences the expression of growth hormone (GH) [18, 19]. GH is directly associated with CRC risk via circulating high levels of insulin-like growth factor I (IGF-I) in the serum. IGF-I has anti-apoptotic and mutagenic properties [20]. Moreover, physical activity is a well-known protective lifestyle factor that modulates circulating IGF-I levels in the serum [20]. Therefore, the current study was designed to assess whether an association exists between physical activity, *PITX1* in CRC risk and to further observe the effect of *PITX1* polymorphism in the relationship between physical activity and CRC risk. However, specific knowledge of whether the interaction between physical activity and genes contributes to the risk of most human cancers is largely unknown.

To date, no information is available regarding the interaction between the physical activity and *PITX1* gene and its effect on CRC risk in a Korean population. Therefore, the objective of the study was to evaluate the association between physical activity, *PITX1* polymorphism and its effect on CRC risk. Further, to investigate the interaction between physical activity, *PITX1* genetic polymorphism in the risk of CRC.

## RESULTS

### General characteristics

Table 1 presents the general characteristics of the study participants with and without CRC. The mean body mass index (BMI) of the controls (24.1 ± 2.7) kg/m^2^ was higher than that of the cases (23.6 ± 3.4) kg/m^2^. The cases were more likely to have a family history of CRC (*p <* 0.001). The cases did less regularly exercise (*p <* 0.001), were less educated (*p <* 0.001), and exhibited lower employment rates (*p <* 0.001), marital statuses (*p <* 0.001) and monthly incomes than the controls (*p <* 0.001). The proportion of non-smokers was similar in the controls and cases (44.3%). The proportion of non-drinkers was similar in the controls (30.3%) and cases (30.2%). The proportion of current drinkers was higher in the controls (60.5%) than in the cases (55.8%) (*p <* 0.001). The physical activity level in terms of MET-minutes per week was higher in controls than cases. The proportion of having high physical activity was higher in controls (*p* = 0.002). The total energy intake was higher in the cases than in the controls (*p <* 0.001).

**Table 1:** General characteristics of the study participants

	Control (*n* = 1846)	Case (*n* = 923)	*P*-value^b^
Age (years)	56.1 ± 9.1	56.6 ± 9.7	>0.999
Gender [*n* (%)]			>0.999
Male	1250 (67.7)	625 (67.7)	
Female	596 (32.3)	298 (32.3)	
Body mass index (kg/m^2^) [*n* (%)]	24.1 ± 2.7	23.6 ± 3.4	<0.001
<25	1215 (66.2)	639 (69.3)	0.103
≥25	620 (33.8)	283 (30.7)	
Smoking status [*n* (%)]			0.156
Non-smoker	818 (44.3)	409 (44.3)	
Former smoker	687 (37.2)	318 (34.5)	
Current smoker	341 (18.5)	196 (21.2)	
Alcohol consumption [*n* (%)]			<0.001
Non-drinker	560 (30.3)	279 (30.2)	
Former drinker	169 (9.2)	129 (14.0)	
Current drinker	1117 (60.5)	515 (55.8)	
First degree family history of colorectal cancer (Yes) [*n* (%)]	99 (5.4)	86 (9.3)	<0.001
Regular exercise [*n* (%)]			<0.001
Yes	1047 (58.2)	311 (33.7)	
No	753 (41.8)	612 (66.3)	
Educational level [*n* (%)]			<0.001
Elementary school or less	127 (7.04)	180 (19.5)	
Middle school	155 (8.6)	141 (15.3)	
High school	587 (32.6)	369 (40.0)	
College or more	934 (51.8)	233 (25.2)	
Occupation [*n* (%)]			<0.001
Group1: Professionals, administrative management, office jobs	481 (26.4)	189 (20.5)	
Group2: Sales and service positions	403 (21.8)	38 (4.1)	
Group3: Agriculture, manufacturing, mining, army service	241 (13.2)	141 (15.3)	
Group4: Housekeeping, unemployment, and others	698 (38.3)	555 (60.1)	
Marital status [*n* (%)]			<0.001
Married	1654 (90.6)	773 (84.1)	
Others (single, divorced, separated, widowed, cohabitating)	171 (9.4)	146 (15.9)	
Monthly income [*n* (%)]^a^			<0.001
<200	388 (23.0)	321 (34.8)	
200–400	754 (44.6)	387 (41.9)	
≥400	545 (32.3)	215 (23.3)	
Physical activity [MET min per week]^c^	2731.0 ± 2945.5	2236.7 ± 2025.3	0.098
Low (<1059)	612 (33.3)	306 (33.2)	0.002
Moderate (1059–2772)	584 (31.8)	348 (37.8)	
High (≥2772)	641 (34.9)	267 (29.0)	
Total energy intake (kcal/day)	1689.6 ± 560.4	2026.3 ± 534.0	<0.001

^a^Unit is 10,000 Won in Korean currency.

^b^*P*-values present the difference between cases and controls. Age, body mass index (continuous) and total energy intake were examined using Student’s *t*-tests; Other variables were assessed using chi-square analysis.

^c^Physical activity [MET min per week] was assessed using Mann-Whitney *U* test.

### The association between physical activity and CRC risk stratified by gender and anatomical site

Table 2 presents the association between physical activity and CRC risk according to gender and anatomical site. Subjects who did regular exercise showed a significantly lower risk of CRC than those who did not regularly exercise (OR = 0.37, 95% CI = 0.30–0.45). Similar associations were observed for colon (OR = 0.38, 95% CI = 0.30–0.49) and rectal (OR = 0.36, 95% CI = 0.28–0.46) cancer. Those in the highest tertile of MET-minutes per week showed a significantly lower risk of CRC than those in the lowest tertile of MET-minutes per week (OR = 0.62, 95% CI = 0.48–0.79, p-trend = < 0.001) even after adjusting for possible confounding variables, including age, gender, marital status, occupation, education, BMI, smoking, alcohol use, family history of CRC and total energy intake. Similar associations were observed for colon and rectal cancer. A significantly lower risk of CRC was observed in males (OR = 0.45, 95% CI = 0.35–0.58) and females (OR = 0.26, 95% CI = 0.18–0.37) who engaged in regular exercise. High physical activity in terms of MET-minutes per week, was associated with a significantly reduced risk of CRC in males (OR = 0.56, 95% CI = 0.41–0.76). However, this association was not significant in females (OR = 0.69, 95% CI = 0.46–1.05), although a reduced risk of CRC was observed.

**Table 2:** The association between physical activity and colorectal cancer risk stratified by gender and anatomical sites

		Colorectal cancer		Colon cancer		Rectal cancer	
	Control (%)	Case (%)	OR (95% CI)^a^	Case (%)	OR (95% CI)^a^	Case (%)	OR (95% CI)^a^
All							
Regular exercise							
No	753 (41.8)	612 (66.3)	1.00	299 (64.6)	1.00	301 (67.8)	1.00
Yes	1047 (58.2)	311 (33.7)	0.37 (0.30–0.45)	164 (35.4)	0.38 (0.30–0.49)	143 (32.2)	0.36 (0.28–0.46)
Physical activity (MET-min/week)							
Low (<1059)	612 (33.3)	306 (33.2)	1.00	152 (32.9)	1.00	146 (33.0)	1.00
Moderate (1059-<2871)	612 (33.3)	366 (39.7)	1.02 (0.81–1.29)	186 (40.3)	1.03 (0.78–1.36)	175 (39.5)	1.03 (0.77–1.37)
High (≥2871)	613 (33.4)	249 (27.0)	0.62 (0.48–0.79)	124 (26.8)	0.63 (0.47–0.85)	122 (27.5)	0.63 (0.46–0.86)
*p*-trend			<0.001		0.001		<0.001
Male							
Regular exercise							
No	489 (40.6)	387 (61.9)	1.00	175 (59.1)	1.00	205 (64.1)	1.00
Yes	716 (59.4)	238 (38.1)	0.45 (0.35–0.58)	121 (40.9)	0.48 (0.35–0.65)	115 (35.9)	0.44 (0.32–0.59)
Physical activity (MET-min/week)							
Low (<1314)	412 (33.2)	215 (34.5)	1.00	100 (33.9)	1.00	110 (34.5)	1.00
Moderate (1314–<3390)	415 (33.4)	239 (38.4)	0.81 (0.61–1.09)	114 (33.4)	0.80 (0.56–1.14)	123 (38.6)	0.83 (0.58–1.18)
High (≥3390)	415 (33.4)	169 (27.1)	0.56 (0.41–0.76)	81 (27.5)	0.56 (0.38–0.82)	86 (27.0)	0.56 (0.39–0.83)
p-trend			<0.001		0.003		0.003
Female							
Regular exercise							
No	264 (44.4)	225 (75.5)	1.00	124 (74.3)	1.00	96 (77.4)	1.00
Yes	331 (55.6)	73 (24.5)	0.26 (0.18–0.37)	43 (25.8)	0.27 (0.17–0.42)	28 (22.6)	0.24 (0.14–0.39)
Physical activity (MET-min/week)							
Low (<735)	198 (33.3)	104 (34.9)	1.00	63 (37.7)	1.00	37 (29.8)	1.00
Moderate (735-<2128)	198 (33.3)	97 (32.6)	0.85 (0.57–1.28)	48 (28.7)	0.67 (0.41–1.09)	48 (38.7)	1.23 (0.73–2.07)
High (≥2128)	199 (33.5)	97 (32.6)	0.69 (0.46–1.05)	56 (33.5)	0.66 (0.41–1.06)	39 (31.5)	0.81 (0.47–1.40)
p-trend			0.086		0.143		0.268

^a^Adjusted for age, gender, marital status, occupation, income, education, BMI, smoking, alcohol, family history of CRC, total energy intake.

### Association between *PITX1* genetic polymorphism and CRC risk based on gender and anatomical site

Table 3 presents the association between the *PITX1* genetic polymorphism and CRC risk based on gender and anatomical site. The *PITX1* genetic variant was significantly associated with an increased risk of CRC, exhibiting an OR (95% CI) of 1.67 (1.16–2.39) for homozygous minor allele carriers (AA) compared with homozygous major allele carriers (CC) after adjusting for potential confounders, including age, gender, education, regular exercise, alcohol use, family history of CRC and total energy intake. The association was similar for colon (OR = 1.71, 95% CI = 1.08–2.70) and rectal (OR = 1.66, 95% CI = 1.03–2.68) cancer. In the dominant model, a significantly increased risk of CRC was observed in carriers with at least one minor allele (AC/AA) compared to that in homozygous major allele carriers (CC) (OR = 1.46, 95% CI = 1.18–1.80). When stratified by gender, a significantly increased risk of CRC was observed in those who carried at least one minor allele (AC/AA) compared to that in male carriers with the major allele (OR = 1.43, 95% CI = 1.10–1.86) and females (OR = 1.51, 95% CI = 1.02–2.24). Similarly significant associations were observed for colon (OR = 1.47, 95% CI = 1.07–2.02) and rectal (OR = 1.39, 95% CI = 1.01–1.92) cancer in males, but the association was significant only for colon cancer in females (OR = 1.59, 95% CI = 1.00–2.53).

**Table 3:** Association between *PITX1* genetic polymorphism and colorectal cancer risk by anatomical sites

All		Colorectal cancer		Colon cancer		Rectal cancer	
	Control (%)	Case (%)	OR (95% CI)^a^	Case (%)	OR (95% CI)^a^	Case (%)	OR (95% CI)^a^
Codominant model							
CC	673 (50.6)	294 (42.8)	1.00	146 (41.8)	1.00	146 (44.4)	1.00
AC	548 (41.2)	321 (46.7)	1.43 (1.15–1.76)	166 (47.6)	1.47 (1.12–1.92)	149 (45.3)	1.34 (1.01–1.77)
AA	110 (8.3)	72 (10.5)	1.67 (1.16–2.39)	37 (10.6)	1.71 (1.08–2.70)	34 (10.3)	1.66 (1.03–2.68)
Dominant model							
CC	673 (50.5)	294 (42.7)	1.00	146 (41.8)	1.00	146 (44.4)	1.00
AC/AA	658 (49.4)	393 (57.2)	1.46 (1.18–1.80)	203 (58.2)	1.50 (1.16–1.95)	183 (55.6)	1.39 (1.06–1.82)
Recessive model							
CC/AC	1221 (91.7)	615 (89.5)	1.00	312 (89.4)	1.00	295 (89.7)	1.00
AA	110 (8.3)	72 (10.5)	1.43 (0.99–2.07)	37 (10.6)	1.41 (0.92–2.19)	34 (10.3)	1.44 (0.92–2.28)
Male							
Codominant model							
CC	474 (52.1)	206 (43.9)	1.00	99 (43.2)	1.00	106 (44.7)	1.00
AC	361 (40.0)	218 (46.5)	1.40 (1.06–1.84)	108 (47.2)	1.44 (1.03–2.01)	108 (45.6)	1.35 (0.96–1.89)
AA	75 (8.2)	45 (9.6)	1.60 (0.99–2.59)	22 (9.6)	1.61 (0.90–2.86)	23 (9.7)	1.61 (0.89–2.88)
Dominant model							
CC	474 (52.1)	206 (43.9)	1.00	99 (43.2)	1.00	106 (44.7)	1.00
AC/AA	436 (47.9)	263 (56.1)	1.43 (1.10–1.86)	130 (56.8)	1.47 (1.07–2.02)	131 (55.3)	1.39 (1.01–1.92)
Recessive model							
CC/AC	835 (91.8)	424 (90.4)	1.00	207 (90.4)	1.00	214 (90.3)	1.00
AA	75 (8.2)	45 (9.6)	1.36 (0.86–2.16)	22 (9.6)	1.35 (0.78–2.34)	23 (9.7)	1.40 (0.80–2.44)
Female							
Codominant model							
CC	199 (47.3)	88 (40.4)	1.00	47 (39.2)	1.00	40 (43.5)	1.00
AC	187 (44.4)	103 (47.3)	1.44 (0.96–2.17)	58 (48.3)	1.54 (0.94–2.50)	41 (44.6)	1.30 (0.77–2.20)
AA	35 (8.3)	27 (12.4)	1.88 (0.97–3.65)	15 (12.5)	1.86 (0.85–4.04)	11 (12.0)	1.79 (0.77–4.14)
Dominant model							
CC	199 (47.3)	88 (40.4)	1.00	47 (39.2)	1.00	40 (43.5)	1.00
AC/AA	222 (52.7)	130 (59.6)	1.51 (1.02–2.24)	73 (60.8)	1.59 (1.00–2.53)	52 (56.5)	1.38 (0.83–2.27)
Recessive model							
CC/AC	386 (91.7)	191 (87.6)	1.00	105 (87.5)	1.00	81 (88.0)	1.00
AA	35 (8.3)	27 (12.4)	1.56 (0.83–2.90)	15 (12.5)	1.48 (0.72–3.08)	11 (12.0)	1.57 (0.71–3.47)

^a^Adjusted for age, gender, education, regular exercise, alcohol, family history of CRC, total energy intake.

### Interaction between regular exercise or physical activity based on MET-minutes per week and *PITX1* genetic variant in the risk of CRC stratified by anatomical site and gender

Table 4 presents the interaction between regular exercise and *PITX1* genetic variant on CRC risk. The interaction was investigated by stratifying patients according to whether they were engaged in regular exercise. Individuals who did not regularly exercise and were major homozygotes were considered the reference group. This polymorphism showed a significantly increased risk of CRC in minor allele carriers who did not regularly exercise (OR = 1.81, 95% CI = 1.37–2.41). Similar associations were observed for male (OR = 1.69, 95% CI = 1.18–2.41) and female (OR = 1.99, 95% CI = 1.23–3.22). Also, a significantly reduced risk of CRC in both groups among homozygous major allele carriers (CC) who did regular exercise (OR = 0.45, 95% CI = 0.32–0.62) and minor allele carriers (AC/AA) who did regular exercise (OR = 0.48, 95% CI = 0.36–0.45) were observed. Among these individuals, a more reduced risk of CRC was observed in major allele carriers who did regular exercise. *PITX1* genetic variant showed a statistically significant interaction with regular exercise and CRC risk in the entire study population (p-interaction = 0.018), and the interaction was marginally significant in females (p-interaction = 0.055). Regarding colon cancer, a significantly increased risk was observed in the group that did not engage in regular exercise and who carried the minor allele compared with the major homozygotes who did not exercise regularly in entire population (OR = 1.95, 95% CI = 1.38–2.75), male (OR = 1.81, 95% CI = 1.17–2.80) and female (OR = 2.13, 95% CI = 1.20–3.78). However, a significant interaction was observed between regular exercise and *PITX1* for colon cancer risk in entire subjects (p-interaction = 0.029). Similar associations were observed for the rectal cancer risk in all subjects (OR = 1.63, 95% CI = 1.17–2.29) and male (OR = 1.59, 95% CI = 1.05–2.40) but a significant interaction was not observed.

**Table 4:** Interaction between regular exercise and *PITX1* genetic variant in the risk of CRC stratified by anatomical sites and gender

Regular exercise	CC		AC/AA		*p*–interaction
	No	Yes	No	Yes	
Colorectal cancer					
All					
No. controls/cases	295/197	373/97	239/265	417/128	
Crude OR (95% CI)	1.00 (ref.)	0.39 (0.29–0.52)	1.66 (1.29–2.13)	0.46 (0.35–0.60)	0.086
Adjusted ORa (95% CI)	1.00 (ref.)	0.45 (0.32–0.62)	1.81 (1.37–2.41)	0.48 (0.36–0.65)	0.018
Male					
No. controls/cases	191/132	278/74	149/169	285/94	
Crude OR (95% CI)	1.00 (ref.)	0.39 (0.27–0.54)	1.64 (1.20–2.24)	0.48 (0.35–0.66)	0.238
Adjusted ORb (95% CI)	1.00 (ref.)	0.45 (0.30–0.66)	1.69 (1.18–2.41)	0.52 (0.36–0.75)	0.173
Female					
No. controls/cases	104/65	95/23	90/96	132/34	
Crude OR (95% CI)	1.00 (ref.)	0.39 (0.22–0.67)	1.71 (1.12–2.61)	0.41 (0.25–0.67)	0.204
Adjusted ORb (95% CI)	1.00 (ref.)	0.45 (0.24–0.83)	1.99 (1.23–3.22)	0.40 (0.23–0.70)	0.055
Colon cancer					
All					
No. controls/cases	295/90	373/56	239/130	417/73	
Crude OR (95% CI)	1.00 (ref)	0.49 (0.34–0.71)	1.78 (1.30–2.45)	0.57 (0.41–0.81)	0.091
Adjusted ORa (95% CI)	1.00 (ref)	0.57 (0.38–0.84)	1.95 (1.38–2.75)	0.38 (0.26–0.56)	0.029
Male					
No. controls/cases	191/57	278/42	149/78	285/52	
Crude OR (95% CI)	1.00 (ref)	0.51 (0.33–0.79)	1.75 (1.17–2.63)	0.61 (0.40–0.93)	0.220
Adjusted ORb (95% CI)	1.00 (ref)	0.58 (0.36–0.94)	1.81 (1.17–2.80)	0.67 (0.43–1.06)	0.149
Female					
No. controls/cases	104/33	95/14	90/52	132/21	
Crude OR (95% CI)	1.00 (ref)	0.46 (0.23–0.92)	1.82 (1.08–3.06)	0.50 (0.27–0.92)	0.254
Adjusted ORb (95% CI)	1.00 (ref)	0.53 (0.25–1.11)	2.13 (1.20–3.78)	0.48 (0.25–0.95)	0.131
Rectal cancer					
All					
No. controls/cases	295/106	373/40	239/128	417/55	
Crude OR (95% CI)	1.00 (ref)	0.30 (0.20–0.44)	1.49 (1.10–2.03)	0.37 (0.26–0.53)	0.478
Adjusted ORa (95% CI)	1.00 (ref)	0.34 (0.22–0.52)	1.63 (1.17–2.29)	0.38 (0.26–0.56)	0.277
Male					
No. controls/cases	191/74	278/32	149/89	285/42	
Crude OR (95% CI)	1.00 (ref)	0.30 (0.19–0.47)	1.54 (1.06–2.24)	0.38 (0.25–0.58)	0.555
Adjusted ORb (95% CI)	1.00 (ref)	0.35 (0.21–0.57)	1.59 (1.05–2.40)	0.41 (0.26–0.65)	0.483
Female					
No. controls/cases	104/32	95/8	90/39	132/13	
Crude OR	1.00 (ref)	0.27 (0.12–0.62)	1.41 (0.82–2.43)	0.32 (0.16–0.64)	0.735
Adjusted ORb	1.00 (ref)	0.33 (0.14–0.79)	1.65 (0.92–2.97)	0.33 (0.16–0.69)	0.550

^a^Adjusted for age, gender, education, alcohol, family history of CRC, total energy intake.

^b^Adjusted for age, education, alcohol, family history of CRC, total energy intake.

Table 5 presents the effect of the interaction between physical activity based on MET-minutes per week and *PITX1* genetic variant on the risk of CRC based on anatomical site and gender. The interaction was investigated by stratifying physical activity according to tertiles of MET-minutes per week. Individuals with a high physical activity who were homozygous for the major allele showed significantly reduced risk of CRC (OR = 0.56, 95% CI = 0.38–0.82) than homozygous major allele carriers with a low physical activity. Subjects who carried the minor allele showed an increased risk of CRC at low and moderate physical activity levels, whereas higher physical activity showed a reduced risk of CRC compared with the reference group. In males, subjects who carried the major allele with high physical activity showed a significantly reduced risk of CRC (OR = 0.34, 95% CI = 0.21–0.54), colon cancer (OR = 0.43, 95% CI = 0.24–0.75) and rectal cancer (OR = 0.25, 95% CI = 0.14–0.46) compared with subjects who carried the major allele and had a low physical activity. No significantly increased risk of CRC was observed in males who carried the minor allele and exhibited different physical activity levels. In contrast, males who carried minor allele and had high physical activity showed significantly reducing risk of CRC (OR = 0.63, 95% CI = 0.41–0.96). However, females who carried the minor allele and exhibited low (OR = 3.10, 95% CI = 1.19–8.06) and moderate (OR = 3.39, 95% CI = 1.39–8.29) levels of physical activity showed a significantly increased risk of colon cancer. There was a significant interaction between physical activity and *PITX1* genetic polymorphism in the risk of colon cancer among female (p interaction = 0.028) but not rectal cancer. In contrast to the male subgroup, females who were homozygous for the major allele with high physical activity exhibited an increased risk of CRC and colon cancer; however, the associations were not significant.

**Table 5:** Interaction between physical activity based on MET-minutes per week and *PITX1* genetic variant in the risk of CRC stratified by anatomical sites and gender

	CC			AC/AA			*P*–interaction
	Low	Moderate	High	Low	Moderate	High	
Colorectal cancer							
All							
No. controls/cases	222/106	213/111	235/77	219/126	229/157	208/108	
Crude OR (95% CI)	1.00 (ref.)	1.09 (0.79–1.51)	0.69 (0.49–0.97)	1.21 (0.88–1.66)	1.44 (1.06–1.95)	1.09 (0.78–1.51)	0.263
Adjusted OR^a^ (95% CI)	1.00 (ref.)	0.97 (0.67–1.40)	0.56 (0.38–0.82)	1.28 (0.90–1.83)	1.29 (0.91–1.82)	0.81 (0.56–1.17)	0.649
Male							
No. controls/cases	162/109	143/48	166/49	159/120	140/65	135/76	
Crude OR (95% CI)	1.00 (ref.)	0.50 (0.33–0.75)	0.44 (0.29–0.66)	1.12 (0.80–1.58)	0.69 (0.47–1.01)	0.84 (0.58–1.21)	0.052
Adjusted OR^b^ (95% CI)	1.00 (ref.)	0.48 (0.30–0.76)	0.34 (0.21–0.54)	1.17 (0.79–1.73)	0.63 (0.41–0.97)	0.63 (0.41–0.96)	0.157
Female							
No. controls/cases	72/17	63/37	64/34	65/27	79/58	78/45	
Crude OR (95% CI)	1.00 (ref.)	2.49 (1.28–4.84)	2.25 (1.15–4.41)	1.76 (0.88–3.52)	3.11 (1.66–5.83)	2.44 (1.28–4.65)	0.284
Adjusted OR^b^ (95% CI)	1.00 (ref.)	2.14 (1.03–4.44)	1.79 (0.84–3.78)	2.06 (0.96–4.41)	3.08 (1.55–6.12)	1.62 (0.79–3.32)	0.091
Colon cancer							
All							
No. controls/cases	222/49	213/53	235/44	219/64	229/84	208/54	
Crude OR (95% CI)	1.00 (ref.)	1.13 (0.73–1.74)	0.85 (0.54–1.33)	1.32 (0.87–2.01)	1.66 (1.12–2.47)	1.18 (0.77–1.81)	0.854
Adjusted OR^a^ (95% CI)	1.00 (ref.)	1.01 (0.64–1.60)	0.69 (0.43–1.12)	1.40 (0.90–2.18)	1.50 (0.98–2.31)	0.89 (0.56–1.41)	0.903
Male							
No. controls/cases	162/49	143/22	166/28	159/53	140/35	135/41	
Crude OR (95% CI)	1.00 (ref.)	0.51 (0.20–0.88)	0.56 (0.33–0.93)	1.10 (0.71–1.72)	0.83 (0.51–1.35)	1.00 (0.63–1.61)	0.148
Adjusted OR^b^ (95% CI)	1.00 (ref.)	0.48 (0.27–0.88)	0.43 (0.24–0.75)	1.14 (0.71–1.85)	0.75 (0.44–1.27)	0.77 (0.46–1.30)	0.165
Female							
No. controls/cases	72/8	63/18	64/21	65/19	79/31	78/23	
Crude OR (95% CI)	1.00 (ref.)	2.57 (1.05–6.32)	2.95 (1.22–7.13)	2.63 (1.08–6.42)	3.53 (1.52–8.18)	2.65 (1.12–6.31)	0.064
Adjusted OR^b^ (95% CI)	1.00 (ref.)	2.12 (0.81–5.53)	2.22 (0.85–5.75)	3.10 (1.19–8.06)	3.39 (1.39–8.29)	1.63 (0.64–4.14)	0.028
Rectal cancer							
All							
No. controls/cases	222/57	213/57	235/32	219/57	229/72	208/53	
Crude OR (95% CI)	1.00 (ref.)	1.04 (0.69–1.57)	0.53 (0.33–0.85)	1.01 (0.67–1.53)	1.23 (0.83–1.82)	0.99 (0.65–1.51)	0.068
Adjusted OR^a^ (95% CI)	1.00 (ref.)	0.91 (0.58–1.43)	0.42 (0.25–0.70)	1.10 (0.70–1.71)	1.08 (0.70–1.66)	0.72 (0.46–1.14)	0.196
Male							
No. controls/cases	162/60	143/26	166/20	159/65	140/30	135/35	
Crude OR (95% CI)	1.00 (ref.)	0.49 (0.29–0.82)	0.33 (0.19–0.56)	1.10 (0.73–1.67)	0.58 (0.35–0.95)	0.70 (0.44–1.13)	0.081
Adjusted OR^b^ (95% CI)	1.00 (ref.)	0.47 (0.27–0.84)	0.25 (0.14–0.46)	1.16 (0.73–1.84)	0.52 (0.30–0.90)	0.50 (0.30–0.85)	0.166
Female							
No. controls/cases	72/9	63/19	64/12	65/6	79/25	78/21	
Crude OR (95% CI)	1.00 (ref.)	2.41 (1.02–5.71)	1.50 (0.59–3.79)	0.74 (0.25–2.19)	2.53 (1.10–5.78)	2.15 (0.93–5.01)	0.472
Adjusted OR^b^ (95% CI)	1.00 (ref.)	2.17 (0.87–5.40)	1.26 (0.47–3.40)	0.87 (0.28–2.69)	2.57 (1.08–6.13)	1.60 (0.65–3.96)	0.648

^a^Adjusted for age, gender, education, alcohol, family history of CRC, total energy intake.

^b^Adjusted for age, education, alcohol, family history of CRC, total energy intake.

Physical activity-All: <1116 (low), 1116-<2925 (moderate), ≥2925 (High)

Physical activity-Male: <1386 (low), 1386-<3465 (moderate), ≥3465 (High)

Physical activity-Female: <660 (low), 660-<2079 (moderate), ≥2079 (High)

## DISCUSSION

In this case-control study involving 2,769 participants (923 cases and 1,846 controls), we observed that there is a significant inverse association between regular exercise and physical activity based on MET-minutes per week in the risk of CRC. Our study observed that *PITX1* rs647161 genetic polymorphism is significantly associated with increased risk of CRC. This significant positive association between *PITX1* genetic variant and CRC was observed for each gender. In addition, *PITX1* rs647161 genetic variant appeared to interact with regular exercise and CRC risk in all subjects and women in particular as well as with colon cancer risk among all subjects in the dominant model. A significant interaction was observed between the physical activity based on MET-minutes per week and *PITX1* genetic polymorphism in the risk of colon cancer among female. CC homozygotes with higher levels of physical activity showed significantly reduced rates of CRC. In contrast, females who were homozygous for the major allele with high physical activity exhibited an increased risk of CRC and colon cancer but not of rectal cancer; however, the associations were not significant. Furthermore, minor allele carriers with low physical activity exhibited a significantly increased risk of colon cancer in the female subgroup. Study results indicated that if the subject carries minor allele and having low physical activity show higher risk for the CRC.

The significant inverse association between physical activity and the risk of CRC observed in the current study is consistent with the results of previous epidemiological studies [21–25]. In the Netherlands, a cohort study on physical activity, occupational sitting time and CRC risk revealed that physical activity was inversely associated with colon cancer, particularly distal colon cancer and rectal cancer, in women; by contrast, these factors were inconsistently associated with CRC in males [26]. Two recent case-control studies focusing on physical activity and CRC risk suggested that the risk of CRC is reduced in individuals with the highest level of physical activity [21, 27]. According to the current study, both men and women who do not engage in regular exercise have an increased risk of CRC, colon and rectal cancers. This association might be explained by the biological hypothesis that increased levels of circulating free IGF-1 and decreased levels of insulin-like growth factor binding protein-3 (IGFBP-3) can increase colon cancer risk [28]. Specifically, in adult women, regular exercise has been associated with decreased levels of circulating estrogen and progesterone. The decreased circulating levels of estrogen, progesterone, serum estradiol, estrone, and androgens are associated with decreased proliferative activity [29]. It is important to note that in our study, we assessed the serum IGF-1 levels in both cases and controls. However, there was no a significant difference of serum IGF-1 between cases and controls (data not shown). Another possible explanation could be that regular exercise has a more extensive effect on gut mobility in the colon than on other tissues [30].

The trend of a reduced risk of CRC with increased physical activity was significant for colon and rectal cancer in males. Inconsistent results were observed regarding rectal cancer risk in females, as there was an increasing risk of rectal cancer associated with moderate level of physical activity. However, the associations and trends were not significant. This result may be attributed to the fact that the number of rectal cancer cases was relatively small in females, consequently leading to limited statistical power. Biological mechanisms related to this difference can be explained by the fact that high intensity exercise stimulates an acute increase in circulating IGF-1 levels, which contributes to cancer growth because high levels of circulating IGF-1 cannot improve the immune response or weight maintenance [28]. In addition, the fact that moderate physical activity does not reduce the risk of rectal cancer in females corresponds with the transit time theory, as the rectum is only intermittently filled with feces and colon peristalsis has a lower influence on the fecal transit time in the rectum [30]. This difference may also be partially explained by genetic factors [31]. Genes associated with GH and IGF metabolism and how physical activity modifies this association must be further examined.

In addition to the above reasons, the calculated MET-minutes per week values might be higher for each stratum due to the overestimation of physical activity in the current study. We categorized physical activity into tertiles based on the distribution of the control group as low, moderate and high. Similarly MET-minutes/week cutoff values were calculated for male and for female as well. A study from National Cancer Institute Consortium which is on leisure time physical activity of moderate to vigorous intensity and mortality: a large pooled cohort analysis [32] have pointed out that a minimum of 7.5 MET-hours/week (450 MET-minutes/week) and twice that level 15 MET-hours/week (900 MET-minutes/week) of physical activity are needed for health benefits and for additional health benefits respectively. In contrast, according to the Inter Act Consortium, they categorized recreational and household activity into quartiles from European Investigation into Cancer and Nutrition-Physical Activity Questionnaire (EPIC-PAQ) based on MET-hours/week for men and women separately. For men, lowest quartile is 32.8 MET-hours/week (1968 MET-minutes/week) and highest quartile is 79.4 MET-hours/week (4764 MET-minutes/week). For women, lowest quartile is 50.1 MET-hours/week (3006 MET-minutes/week) and highest quartile is 93.5 MET-hours/week (5610 MET-minutes/week) [33].

*PITX1*, a bicoid-related homeobox gene, is expressed preferentially in the developing anterior pituitary gland [34] and encodes PITX1, a bicoid class homeodomain protein that functions as a transcription factor [19, 35]. PITX1 expression can promote the expression of GH in the anterior pituitary gland [19] and the PITX1 transcription factor is necessary for the activation of GH [34]. Some previous studies have also shown that GH is involved in regulating circulating serum levels of IGF-1 [36–39], and it is believed that GH is the main stimulator and metabolic mediator of IGF-1 synthesis and release [35]. This mechanism suggests that *PITX1* can indirectly modulate serum levels of IGF-1. Furthermore, a review of the influence of exercise on the IGF axis on an oncological, physiological basis has demonstrated that physical activity can modulate serum levels of IGF-1 [40]. IGF should be considered in terms of local (in tissue) and peripheral (in serum) levels. Local levels of IGF-1, such as in muscle tissues, consistently increases after exercise regardless of the length and intensity of the exercise. However, the results for peripheral levels of IGF-1 have been more differential (increases, decreases or no changes) [28]. The effects of physical activity on metabolizing hormone levels and genetic factors may influence the risk of colon cancer more than that of rectal cancer [41]. An updated, systematic review and meta-analysis on circulatory IGF peptides and CRC risk showed that elevated levels of systemic IGF-1 are associated with an increased CRC risk [42]. Moreover, IGF-1 exhibits anti-apoptotic and mutagenic properties in CRC carcinogenesis [43, 44]. Therefore, we hypothesized that there is an association between physical activity, *PITX1* and CRC risk; thus, we assessed the interaction between physical activity and *PITX1* genetic polymorphism in CRC risk.

The present study demonstrates a significant interaction between regular exercise, *PITX1* rs647161 in the risk of CRC and colon cancer. Subjects who carried the minor allele, and did not regularly exercise showed a significantly higher risk of CRC, colon cancer, and rectal cancer. A recent study assessed gene-environment interactions involving recently identified CRC susceptibility loci and found that the association between those identified genetic susceptibility loci, including rs647161, and CRC were not modified by several environmental factors, such as BMI, alcohol, smoking and various other dietary factors [14]. Regarding the interactions between physical activity based on MET -minutes per week, *PITX1* genetic variant in the risk of CRC, no significant interactions were observed even in the stratified analysis by gender and anatomical site. This result may have been due to the limited power to detect an interaction. Interestingly a significantly increased risk of colon cancer was detected in female participants who carried a minor allele and had low physical activity level. Further, the interaction between physical activity and *PITX1* genetic variant in the risk of colon cancer was observed in female. The increased risk of colon cancer among females who carried the risk allele can be explained by the increased levels of circulating IGF-1, which can promote cancer cell growth and inhibit apoptosis [28]. A study of the role of insulin and IGF-1 in energy balance and cancer showed that the expression of genes related to the IGF pathway is modified by the absence of physical activity [45]. Although there is a paucity of data regarding the interaction between physical activity and *PITX1*, our findings and evidence may guide future prevention strategies. Moreover, understanding the interaction between *PITX1* and physical activity will help to elaborate underlying biological mechanisms of cancer.

Due to the widespread availability of genetic data in epidemiologic research, GWAS have identified several SNPs associated with CRC risk. The *PITX1* gene encodes pituitary homeobox transcription factor 1 and is one of the three members of the *PITX1* family. *PITX1* and other family members are involved in organ development and left-right asymmetry [46, 47]. According to a post- GWAS study [13] and genome-wide meta-analysis [48], the *PITX1* gene functions as a tumor suppressor gene related to CRC carcinogenesis. Different tissues, including bone, cartilage, and muscle, and fibroblast cells express *PITX1* in addition to the anterior pituitary gland. *PITX1* is also a master regulator of hind-limb identity [13]. However, the role of *PITX1* in carcinogenesis remains controversial. A study assessing the involvement of *PITX1* in human cancer demonstrated that, *PITX1* expression is reduced in gastric, bladder and colon cancers relative to that of normal tissues and concluded that *PITX1* is likely a relevant tumor suppressor gene [49]. To date, approximately 80 molecular markers have been reported to be correlated with performance, physical exercise, and fitness [37, 50]. One study based on the joint identification of genetic variants associated with physical activity in a Korean population identified no significant SNP at the genome-wide level via single SNP association tests; however, the joint identification of SNPs revealed multiple SNPs with good predictive power for physical activity [51]. Nonetheless, it is important to emphasize that there is paucity of information regarding the interaction between physical activity and *PITX1* genetic polymorphism.

No study has reported the interaction between *PITX1* polymorphism, physical activity and CRC risk. However, this study assessed whether the *PITX1* polymorphism can modify the association between physical activity and CRC risk in our study population. To the best of our knowledge, this is the first study to provide new epidemiological evidence of the role of the *PITX1* genetic polymorphism in the association between physical activity and CRC carcinogenesis. Another strength of the study involves the analysis of the interaction effect of physical activity, CRC risk and the *PITX1* genetic polymorphism depending on the anatomical sub-sites in each gender because several etiological factors may vary among the sub-sites [42]. For instance, certain physiological, molecular, and biochemical differences in carcinogenesis have been observed at the sub-sites [52]. In addition, epidemiologic differences in cancers at specific sub-sites and between genders have been examined. Thus, such anatomical and gender stratifications may be useful for future research efforts in this area and may further our understanding about cancer risk and prevention.

However, this study has some potential limitations. First, this study was conducted as a case-control study. The controls were recruited from those who voluntarily participated in a health screening program in Korea. Thus, the possibility of concern about a health-related behavior in these controls may be associated with a reduced risk of CRC. Second, the recall of regular exercise and physical activity habits may differ between the cases and controls or between males and females due to different levels of health and behavior compliance. In addition, the cases are more prone to recall by emphasizing their physical inactivity behavior. Third, although we adjusted for numerous potentially confounding factors, the effects of other residual confounding factors, such as GH level and serum IGF-I level, which exhibit a strong biological plausibility among the *PITX1* gene, physical activity and CRC carcinogenesis, were not taken into account. Fourth, our sample size was not large enough to perform genetic analysis based on anatomical sites and gender; thus, we did not have sufficient power to detect a small interaction effect. Finally, only one SNP of the *PITX1* gene was evaluated which may not represent the entire gene.

In conclusion, our study identified an inverse association between physical activity and CRC risk in the entire study population. Furthermore, an interaction between regular exercise, CRC risk and the *PITX1* polymorphism was observed and was dependent on the anatomical sub-site. Individuals with the *PITX1* polymorphism exhibit an increased risk of CRC if they do not regularly exercise. Thus, individuals in the Korean population who carry minor allele can have benefit from physical activity regarding prevention of CRC risk. Finally, future larger studies with an increased number of genetic variants are warranted to observe the various effects in individuals.

## MATERIALS AND METHODS

### Study participants

**Figure 1:**
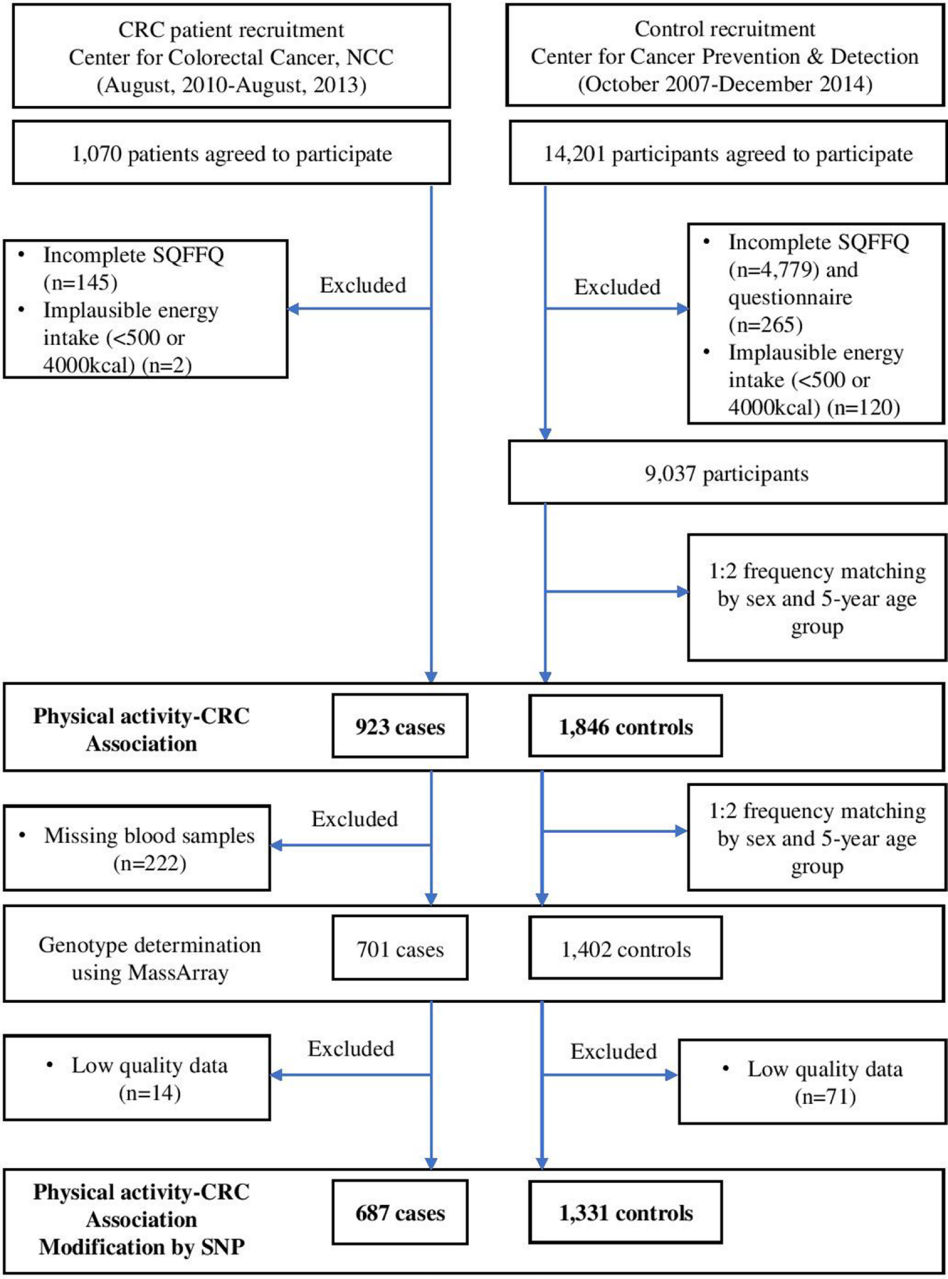
Simplied flow chart for the selection of study subject

Cases included patients who were newly diagnosed with CRC between August 2010 and August 2013 at the Center for Colorectal Cancer of the NCC in Korea. Of the 1,070 patients who agreed to participate in this study and provided informed consent, 145 were excluded due to incomplete semi-quantitative food frequency questionnaires (SQFFQ), and two patients were excluded due to implausible energy intakes (< 500 kcal/day or > 4000 kcal/day). Therefore, 923 patients were included in the analysis. The controls were selected from a group of participants who visited the Center of Cancer Prevention and Detection at the same hospital between October 2007 and December 2014 for a health check-up provided by the National Health Insurance Cooperation, which covers the entire Korean population. Of the 14,201 participants who agreed to participate in this study, 5,044 were excluded due to incomplete SQFFQ data, and 120 were excluded due to implausible energy intakes. Of the remaining 9,037 participants, random frequency matching of two controls per case was performed based on gender and 5-year age group. Therefore, 923 cases and 1,846 controls were selected for the analysis of the association between physical activity and CRC risk. Of these participants, 701 cases and 1,402 controls were selected, and their genotypes were determined at a 1:2 frequency and matched by sex and 5-year age group. Finally, 687 cases and 1,331 healthy controls with qualifying genotype data were selected for the analysis of the genetic association (Figure 1: a simplified flow chart describing the selection of study subjects). All participants provided written informed consent prior to participation. The study protocol was approved by the Institutional Review Board (IRB) of the NCC (IRB Nos. NCCNCS-10-350 and NCC2015-0202). All study protocols were performed according to the guidelines and regulations of the IRB of the NCC.

### Data collection

Information regarding the participants’ socio-demographic characteristics (e.g., age, education, smoking, alcohol drinking, regular exercise, occupation, household income, and marital status) was collected using a structured questionnaire. Each participant’s habitual dietary intake was assessed using a 106-item SQFFQ. The validity and reproducibility of the questionnaire were previously reported [53]. Individual energy and food intake were calculated using CAN-PRO 4.0 (Computer Aided Nutritional Analysis Program, The Korean Nutrition Society, Seoul, Korea). Physical activity was measured using the short version of the International Physical Activity Questionnaire (IPAQ). The information was summarized into metabolic equivalents of task (MET) units of minutes per week [54].

### Genotyping

Genotyping of the *PITX1* rs647161 C>A polymorphism was conducted as follows. Genomic DNA was extracted using the MagAttract DNA Blood M48kit (Qiagen, Hilden, Germany) and BioRobot M48 automatic extraction equipment (Qiagen), according to the manufacturers’ instructions. Genotyping was performed using the MassArray iPLEX gold assay (Agena Bioscience, San Diego, CA, USA).

### Statistical analysis

To compare the demographic and lifestyle characteristics between the cases and controls, Chi-square test and Student’s *t*-test were performed for categorical variables and continuous variables, respectively. To investigate the association between physical activity and CRC risk, physical activity was categorized into tertiles according to the distribution of MET minutes per week in the control group. The lowest tertile of MET-minutes per week was used as the reference category. The odds ratio (OR) and 95% confidence intervals (CIs) were estimated using unconditional logistic regression models. The multivariable model was adjusted for age, gender, marital status, occupation, education, BMI, smoking, alcohol use, family history of CRC and total energy intake. The median MET minutes per week in each tertile was used as a continuous variable to test for trends. A subgroup analysis was performed after stratification by gender and anatomical location. A multinomial logistic regression model was used for the stratified analysis based on the anatomical sub-sites (colon and rectum).

Chi-square test was used to test for Hardy-Weinberg equilibrium (HWE) for *PITX1* rs647161 in the control group. The association between the *PITX1* genetic polymorphism and CRC risk was observed in three models, i.e., co-dominant, dominant, and recessive. The OR and 95% CI were estimated using both crude and multivariable logistic regression models. Furthermore, the interaction terms were examined to investigate whether the *PITX1* genetic variant modifies the association between physical activity and CRC risk. Regular exercise was considered as two categories noting whether the participants engage in regular exercise or not. The subjects who were homozygous for the major allele and did not engage in regular exercise were considered the reference. Physical activity was then categorized into three levels based on MET minutes per week (i.e., low, moderate, high), and subjects homozygous for the major allele with a low physical activity were considered the reference. The test for interactions between the physical activity and *PITX1* genetic polymorphism in relation to CRC were conducted using logistic regression models via likelihood ratio tests. All statistical analyses were performed using SAS version 9.4 (SAS Institute Inc., Cary, NC, USA). A two-sided *p*-value of less than 0.05 was considered statistically significant.

## Abbreviations

BMI: Body mass index
CI: Confidence interval
CRC: Colorectal cancer
EPIC-PAQ: European Investigation into Cancer and Nutrition-Physical Activity Questionnaire
GH: Growth hormone
GWAS: Genome-wide association study
HWE: Hardy Weinberg equilibrium
IGF-I: Insulin-like growth factor-I
IGFBP-3: Insulin-like growth factor binding protein-3
IPAQ: International physical activity questionnaire
IRB: Institutional review board
MET: Metabolic equivalents of task
NCC: National cancer center
OR: Odd ratio
PITX1: Paired-like homeodomain 1
SNPs: Single nucleotide polymorphisms
SQFFQ: Semi-quantitative food frequency questionnaire
