# Correction: Interaction between physical activity, *PITX1* rs647161 genetic polymorphism and colorectal cancer risk in a Korean population: a case-control study

**DOI:** 10.18632/oncotarget.26227

**Published:** 2018-10-09

**Authors:** Madhawa Neranjan Gunathilake, Jeonghee Lee, Young Ae Cho, Jae Hwan Oh, Hee Jin Chang, Dae Kyung Sohn, Aesun Shin, Jeongseon Kim

**Affiliations:** ^1^ Department of Cancer Control and Population Health, Graduate School of Cancer Science and Policy, Goyang-si, Gyeonggi-do 10408, South Korea; ^2^ Department of Biomedical Science, Graduate School of Cancer Science and Policy, Goyang-si, Gyeonggi-do 10408, South Korea; ^3^ Center for Colorectal Cancer, National Cancer Center Hospital, National Cancer Center, Goyang-si, Gyeonggi-do 10408, South Korea; ^4^ Department of Preventive Medicine, Seoul National University College of Medicine, Jongno-gu, Seoul 03080, South Korea

**This article has been corrected:** The correct Funding information is given below:

**FUNDING**

This work was supported by grants from the National Cancer Center, Korea (1630960, 1710882, and 1810090), the National Research Foundation of Korea (2015R1A5A6001906, 2010-0010276, and 2013R1A1A2A10008260), and the fund from the Ministry of Food and Drug Safety (17162MFDS040).

Original article: Oncotarget. 2018; 9:7590-7603. https://doi.org/10.18632/oncotarget.24136

